# The complete genome, structural proteome, comparative genomics and phylogenetic analysis of a broad host lytic bacteriophage ϕD3 infecting pectinolytic *Dickeya* spp.

**DOI:** 10.1186/s40793-015-0068-z

**Published:** 2015-09-24

**Authors:** Robert Czajkowski, Zofia Ozymko, Joanna Siwinska, Adam Ossowicki, Victor de Jager, Magdalena Narajczyk, Ewa Łojkowska

**Affiliations:** Department of Biotechnology, Intercollegiate Faculty of Biotechnology, University of Gdansk and Medical University of Gdansk, Kladki 24, 80-822 Gdansk, Poland; Netherlands Institute of Ecology (NIOO-KNAW), Wageningen, The Netherlands; Laboratory of Electron Microscopy, Faculty of Biology, University of Gdansk, Gdansk, Poland

## Abstract

Plant necrotrophic *Dickeya* spp. are among the top ten most devastating bacterial plant pathogens able to infect a number of different plant species worldwide including economically important crops. Little is known of the lytic bacteriophages infecting *Dickeya* spp. A broad host lytic bacteriophage ϕD3 belonging to the family *Myoviridae* and order *Caudovirales* has been isolated in our previous study. This report provides detailed information of its annotated genome, structural proteome and phylogenetic relationships with known lytic bacteriophages infecting species of the *Enterobacteriaceae* family.

## Introduction

Pectinolytic *Dickeya* spp. can cause disease on a number of arable and ornamental crops worldwide including potato, tomato, carrot, onion, pineapple, maize, rice, hyacinth, chrysanthemum and calla lily resulting into severe economic losses [1]. *Dickeya* spp. are recognized to be among the top ten most important bacterial pathogens in agriculture [2]. To date there is no effective control of *Dickeya* spp. in agriculture due to the lack of practical measures and strategies [3].

Lytic bacteriophages have been proposed as potential biological control agents against various pathogenic bacterial species including plant pathogens [4]. Their potential to control plant bacterial diseases has been evaluated among others against *Erwinia amylovora*, *Xanthomonas**pruni*, *Ralstonia solanacearum* and also were experimentally tested against *Pectobacterium* spp. and *Dickeya* spp. in different crop systems [4]. In the case of *Pectobacterium* spp. and *Dickeya* spp. lytic bacteriophages, only limited attempts have been made so far to isolate and characterize these bacteriophages in detail [5, 6] and to provide information on their genomes and structural proteomes [7].

At present, only two *Dickeya* spp. lytic bacteriophages: LimeStone1 and ϕD5 were characterized in detail, *viz*. their complete genomes are available in the Genbank (accessions: NC019925 and KJ716335, respectively) and information on other features (e. g. structural proteomes and host range, multiplicity of infection and adsorption to bacterial hosts) is also available [6, 7].

### Virus information

**Fig. 1:**
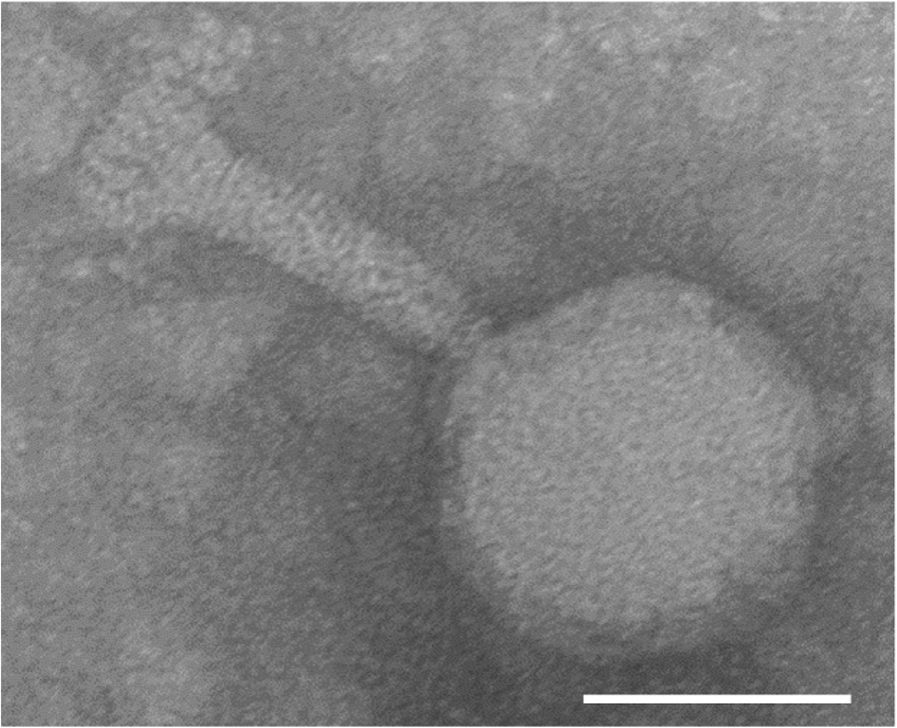
Transmission electron micrograph of *Dickeya* spp. bacteriophage ϕD3 stained with uranyl acetate. Bacteriophage particle was purified four times by passaging individual plaques using the soft top agar method and *D. solani* IPO2222 as a host. Phage suspension of ca. 10^5^ plaque forming units (pfu) ml^−1^ in 1/4 Ringer’s buffer was used for microscopy. At least 10 different photographs were taken. The micrograph presents typical ϕD3 phage particle. Bar marker represents 100 nm [5]

Bacteriophage ϕD3 was isolated from garden soil collected in Kujawsko-Pomorskie region (Kuyavian-Pomeranian Province) in 2013 in Poland and it has been characterized in full for morphologic and phenotypic features [5]. It is a broad host lytic phage belonging to *Myoviridae* family and *Caudovirales* order and infecting isolates of *D. solani*, *D. dadantii*, *D. dianthicola*, *D. zeae* and *D. chrysanthemi* species. In transmission electron microscopy, this bacteriophage was characterized by the presence of a 130 nm long contractile tail, a head of 100 nm in diameter and of dodecahedral symmetry [5] (Fig. 1).

### Chemotaxonomic data

**Fig. 2:**
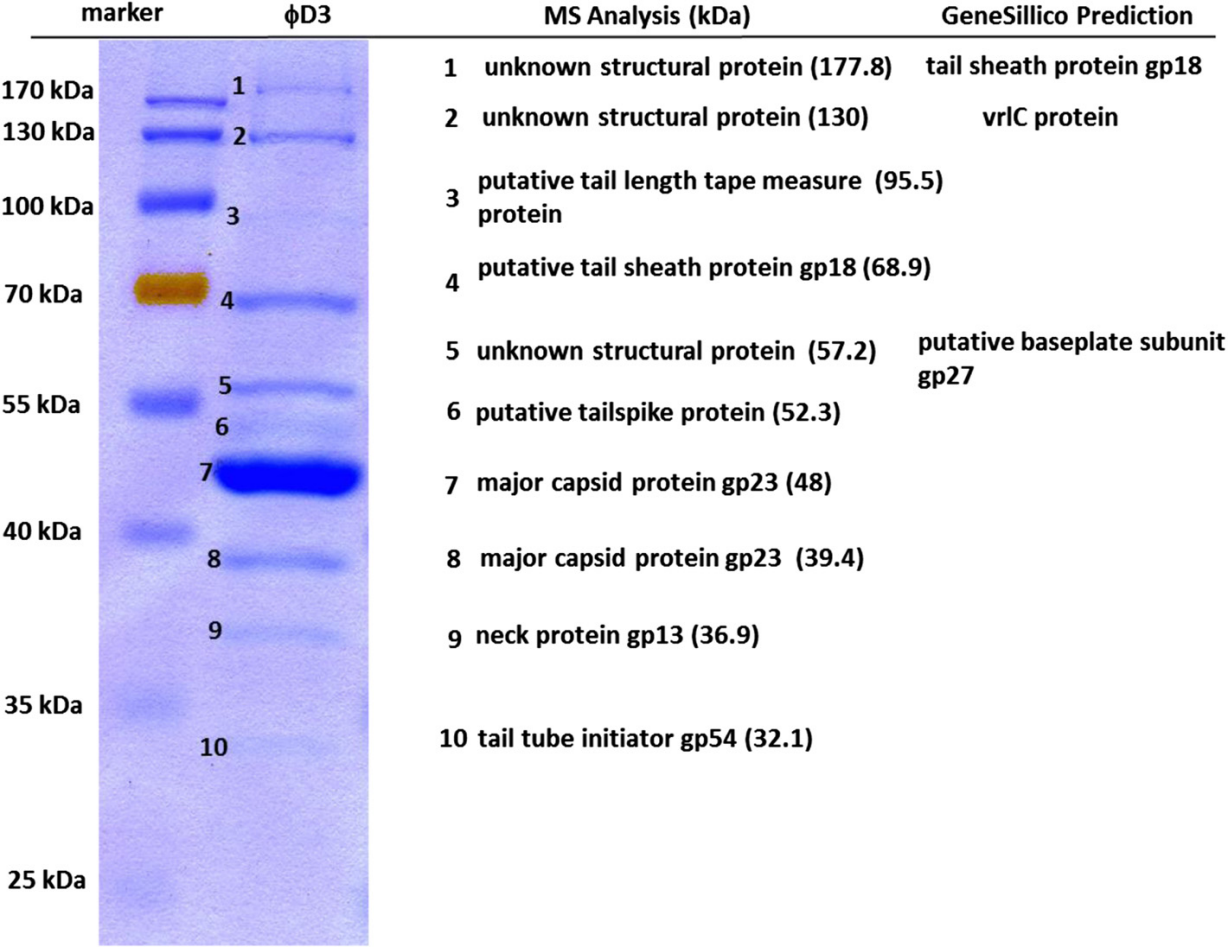
SDS-PAGE and MS analysis of ϕD3 structural proteins. For SDS-PAGE electrophoresis ca. 10^9^ pfu ml^−1^ were mixed with Laemmli buffer and frozen in liquid nitrogen for 1-2 min. following the boiling at 95 °C for 5 min. The phage proteins were separated in 12 % acrylamide SDS-PAGE gel for ca. 19 h t 50 V at 22 °C. The bands were stained with PageBlue Coomasie Blue (Thermo Scientific) according to protocol provided by the manufacturer. For MS analysis of phage structural proteins, protein bands obtained from SDS-PAGE were excised from gel with a sterile scalpel and sent to the mass spectrometry analysis to Mass Spectrometry Laboratory, Institute of Biochemistry and Biophysics, Polish Academy of Science in Warsaw, Poland. Possible molecular functions of the unknown structural proteins were elucidated using Gene Sillico Protein Structure Prediction Meta-server [9]

To better characterize bacteriophage ϕD3, we performed in addition to the genome characterization also SDS-PAGE and MS analysis of its structural proteins [8]. Protein bands were excised from the gels with a sterile scalpel and used for mass spectrometry analysis performed at the Mass Spectrometry Laboratory, Institute of Biochemistry and Biophysics, Polish Academy of Sciences in Warsaw, Poland. In order to predict the molecular functions of the unknown structural proteins obtained from SDS-PAGE and MS analysis we used GeneSillico Protein Structure Prediction Meta-server containing known three-dimensional (3D) protein structures [9] and PSI-BLAST accessed *via* NCBI website [10]. The computational protein predictions with the highest scores were considered as the most valid [9, 10]. This direct and bioinformatic approach led to the experimental identification of 10 structural proteins of ϕD3. From these, the function of 7 proteins could be assigned directly based on sequence similarities with the other known phage proteins (Fig. 2). The most abundant protein was major capsid protein gp23. Three proteins present in the ϕD3 proteome were characterized by MS as unknown structural proteins for which no function could be inferred based on homology with amino acid sequences present in the current databases. These proteins were analyzed by comparing their sequences with protein sequences deposited in the GeneSillico protein 3D structure database. We were then able to assign functions to all unknown proteins using this approach.

## Genome sequencing information

### Genome project history

A number of recent studies have shown that bacteriophages play a substantial role in global ecosystems and have a direct bearing on the ecology and evolution of their hosts. The ϕD3 genome is the third (after LimeStone1 and ϕD5) complete genome of lytic bacteriophage virulent to plant pathogenic *Dickeya* spp. available to the scientific community. Genome sequencing and analysis provide a better possibility to deduce phage infections in host cells and phage interaction with a variable environment. This genome project was deposited in NCBI Genbank as Bioproject PRJNA242299 under the title: “Bacteriophages of *Pectobacterium* spp. and *Dickeya* spp. Genome sequencing”. A summary of the project information is shown in Table 2.

### Growth conditions and genomic DNA preparation

*D. solani*IPO2222 (type strain for *D. solani*), grown on tryptone soya agar (Oxoid) and/or in tryptone soya broth (Oxoid), was used in all experiments as a ϕD3 host. Bacteriophage ϕD3 was isolated as described previously [5] from *Dickeya* spp.-free garden soil which may indicate that the phage can infect also different soil-borne bacteria as additional hosts. Purification and concentration of phage particles followed the previous protocols and included: DNase I and RNase A treatments, CsCl gradient ultracentrifugation and dialysis to remove CsCl from phage concentrated samples [7]. Purified phage particles were resuspended in 500 μl of 5 mM MgSO_4_ or in 1/4 Ringer’s buffer (Merck) and stored at 4 °C in the dark. The ϕD3 genomic DNA was purified using CTAB method as described in [11].

### Genome sequencing and assembly

The genome was sequenced using the Illumina next generation technology at Baseclear, The Netherlands, following the manufacturer’s instructions (Illumina). The sequencing library yielded ca. 270 Mb clean data reads after sets of rigorous filtrations against bacterial host genomic DNA (*D. solani* strain IPO2222, Genbank accession: AONU00000000). *De novo* assembly of the ϕD3 genome from the resulting raw reads was performed using CLC Genomic Workbench 7.5 (CLC bio) as described earlier [12] which provided >1500 x coverage of the genome.

### Genome annotation

The ϕD3 genome was mapped and annotated using available bacteriophage genomic sequences deposited in GenBank. Structural and functional annotations for the ϕD3 genome were obtained from the Annotation Service Automatic Pipeline (Institute for Genome Science, School of Medicine, University of Maryland, USA) and confirmed using RAST set to auto settings. Additional analysis of the gene predictions and annotations was supplemented using Manatee accessed *via* the website of IGS, University of Maryland, USA. The lifestyle of ϕD3 (temperate [lysogenic] or lytic) was predicted using PHACTS [13]. To find potential genes acquired by ϕD3 coding for toxins and allergens, the genome sequence was analyzed bioinformatic analysis using Virulence Finder 1.2 and VirulentPred.

### Genome properties

**Fig. 3:**
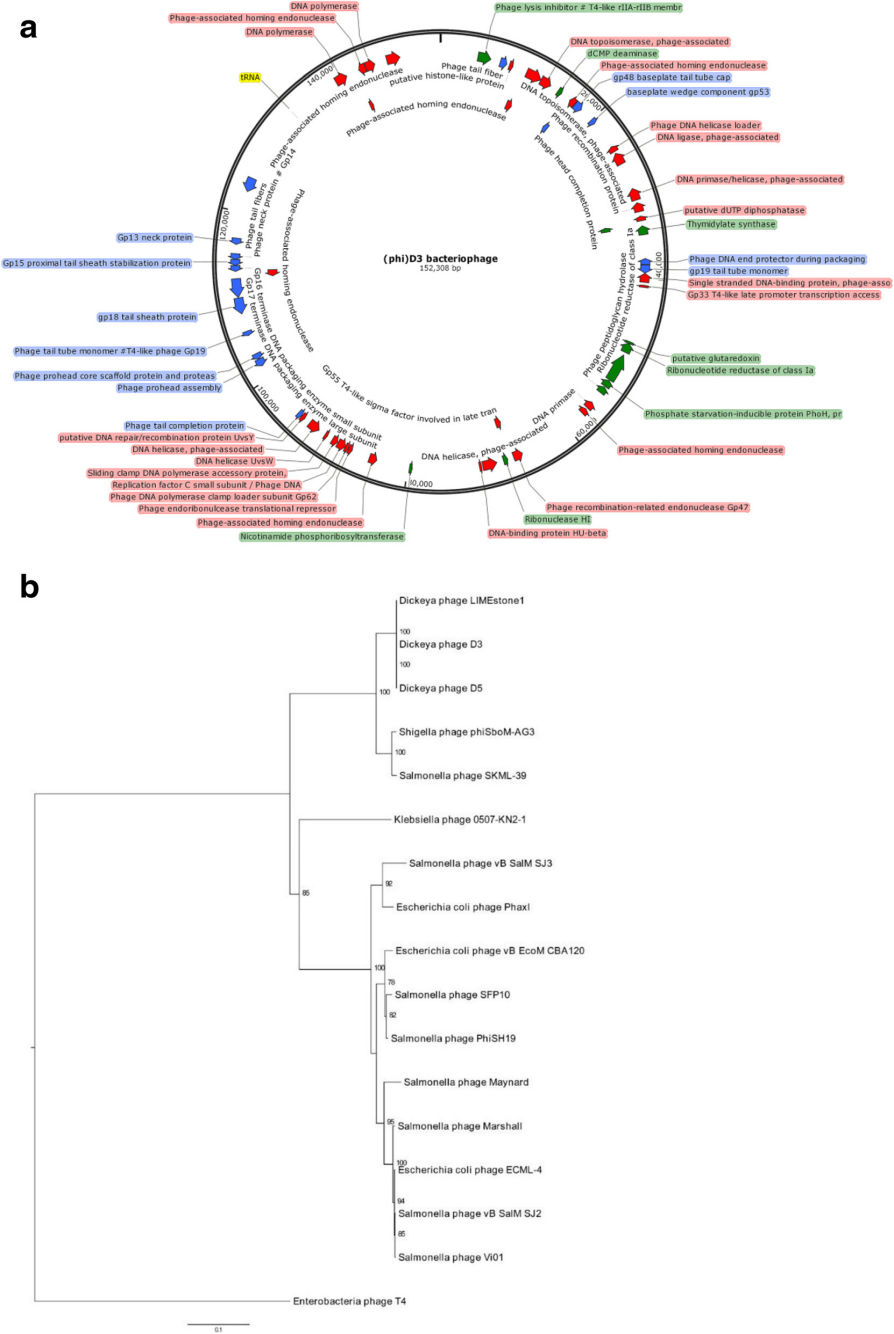
Phage ϕD3 genome (**a**) and phylogenetic analysis (**b**). **a** The genome of bacteriophage ϕD3 (152,308 bp). Structural and functional annotations were obtained from IGS Annotation Service (http://ae.igs.umaryland.edu/cgi/index.cgi) and from RAST (http://rast.nmpdr.org/). ORFs coding for proteins involved in DNA metabolism, transcription and translation are marked in red, ORFs coding for proteins involved in phage particle assembly are marked in blue and ORFs coding for enzymes are marked in green. Arrows indicate the direction of transcription and translation. The ORFs coding for hypothetical proteins are not shown on the map. The figure was generated using a genome visualization tool – SnapGene ver. 2.6.2. **b** Maximum likelihood tree based on the aligned consensus nucleotide sequences (600 bp. long each) of gp20 genes of bacteriophages closely related to *Dickeya* sp. phage ϕD3. *Enterobacteria* phage T4 was used as an outgroup. Phylogenetic studies were performed using Phylip package. Bootstrap values (per 1000 replicates) are shown at branch points. The bar indicates the number of substitutions per sequence position

Tables 1, 2, 3 and 4 summarize the properties and statistics of the ϕD3 genome. The capsid of ϕD3 contains circular double-stranded DNA genome of 152 308 bp, with an average GC content of 49.3%. The complete genome possesses 191 open reading frames (190 ORFs with the average gene length calculated to be 730 nucleotides) and one tRNA-Met (tRNA-methionine) ORF. A total of 105 ORFs (54.9%) have assigned function, whereas 45.1% (86 ORFs) are conserved hypothetical ORFs for which no homology with known genes was found in the NCBI database. Forty one ORFs (21.5%) were unclassified with no assigned role category (Fig. 3a). The lifestyle of ϕD3 predicted from PHACTS indicated that it is a lytic bacteriophage. The ϕD3 genome does not contain any genes coding for (known) toxins, allergens and other virulence factors as tested by VirulenceFinfer 1.2 and VirulencePred. Likewise, a search in BLAST did not reveal the presence of toxins, allergens, integrases and/or antibiotic resistance genes in the genome of ϕD3. The compete genome sequence of ϕD3 was deposited at DDBJ/EMBL/Genbank under accession number KM209228.

**Table 1:** Classification and general features of *Dickeya* spp. bacteriophage ϕD3

MIGS ID	Property	Term	Evidence code^a^
	Classification	Domain: Viruses, dsDNA viruses, no RNA viruses	TAS [5]
		Phylum: unassigned	TAS [5]
		Class: unassigned	TAS [5]
		Order: *Caudovirales*	TAS [5]
		Family: *Myoviridae*	TAS [5]
		Genus: unassigned	TAS [5]
		Species: unassigned	TAS [5]
	Gram stain	Not applicable	TAS [5]
	Particle shape	Icosahedral	IDA
	Motility	Not applicable	TAS [5]
	Sporulation	Not applicable	TAS [5]
	Temperature range	Not applicable	TAS [5]
	Optimum temperature	Not applicable	TAS [5]
	pH range; Optimum	Not applicable	TAS [5]
	Carbon source	Not applicable	TAS [5]
MIGS-6	Habitat	Soil	IDA
MIGS-6.3	Salinity	Not applicable	TAS [5]
MIGS-22	Oxygen requirement	Not applicable	TAS [5]
MIGS-15	Biotic relationship	Obligate intracellular parasite of *Dickeya* spp.	IDA
MIGS-14	Pathogenicity	Lytic virus of *Dickeya* spp.	IDA
MIGS-4	Geographic location	Poland / Kujawsko-Pomorskie (Kuyavian-Pomeranian Province)	IDA
MIGS-5	Sample collection	February 18, 2013	IDA
MIGS-4.1	Latitude	53.68 N	IDA
MIGS-4.2	Longitude	18.09 E	IDA
MIGS-4.3	Depth	20 cm	IDA
MIGS-4.4	Altitude	118 m	IDA

^a^Evidence codes - *IDA* inferred from direct assay, *TAS* traceable author statement (i.e., a direct report exists in the literature), *NAS* non-traceable author statement (i.e., not directly observed for the living, isolated sample, but based on a generally accepted property for the species, or anecdotal evidence). These evidence codes are from the Gene Ontology project [20]

**Table 2:** Project information

MIGS ID	Property	Term
MIGS-31	Finishing quality	Complete
MIGS-28	Libraries used	One paired-end library
MIGS-29	Sequencing platforms	Illumina
MIGS-31.2	Fold coverage	1753×
MIGS-30	Assemblers	CLC Genomics Workbench, version 7.0.3
MIGS-32	Gene calling method	RAST version 4.0, IGS Annotation Service (Manatee)
	Locus Tag	HQ80
	Genbank ID	KM209228
	GenBank Date of Release	16.07.2016 (earlier upon publication)
	GOLD ID	GP0111934
	BIOPROJECT	PRJNA242299
MIGS-13	Source Material Identifier	NCNRC002.D3
	Project relevance	Biological effects in soil and plant environments

**Table 3:** Genome statistics

Attribute	Value	% of Total
Genome size (bp)	152,308	100.0
DNA coding (bp)	138,905	91.1
DNA G + C (bp)	75,088	49.3
DNA scaffolds	1	100.0
Total genes	191	100.0
Protein coding genes	190	99.5
RNA genes	1	0.5
Pseudo genes	0	0.0
Genes in internal clusters	0	0.0
Genes with function prediction	105	54.9
Genes assigned to COGs	64	33.5
Genes with signal peptides	0	0.0
Genes with transmembrane helices	0	0.0

**Table 4:** Number of genes associated with general COG functional categories

Code	Value	% age	Description
J	0	0.00	Translation, ribosomal structure and biogenesis
A	1	0.53	RNA processing and modification
K	4	2.11	Transcription
L	9	4.74	Replication, recombination and repair
B	0	0.00	Chromatin structure and dynamics
D	6	3.16	Cell cycle control, Cell division, chromosome partitioning
V	0	0.00	Defense mechanisms
T	0	0.00	Signal transduction mechanisms
M	0	0.00	Cell wall/membrane biogenesis
N	0	0.00	Cell motility
U	0	0.00	Intracellular trafficking and secretion
O	0	0.00	Posttranslational modification, protein turnover, chaperones
C	1	0.53	Energy production and conversion
G	0	0.00	Carbohydrate transport and metabolism
E	0	0.00	Amino acid transport and metabolism
F	2	1.05	Nucleotide transport and metabolism
H	0	0.00	Coenzyme transport and metabolism
I	0	0.00	Lipid transport and metabolism
P	0	0.00	Inorganic ion transport and metabolism
Q	0	0.00	Secondary metabolites biosynthesis, transport and catabolism
R	41	21.6	General function prediction only
S	10	5.3	Function unknown
-	139	60.98	Not in COGs

The total is based on the total number of protein coding genes in the genome

### Comparisons with other genomes of *Dickeya* spp. bacteriophages and bacteriophage T4

**Fig. 4:**
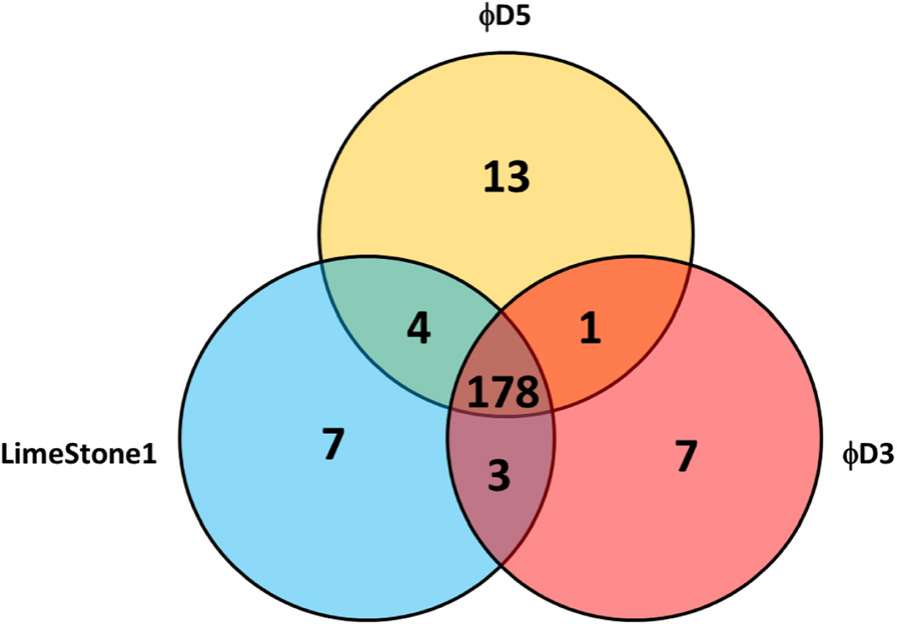
Core genome of ϕD3, ϕD5 and LimeStone1 bacteriophages

Multiple genome alignment was performed using Mauve [14] and comparative genomics analysis was done using EDGAR [15]. A pairwise comparison of the complete four genome sequence of ϕD3, ϕD5 [7], LimeStone1 [6] and *Enterobacteriaceae* bacteriophage T4 revealed that ϕD3, ϕD5 and LimeStone1 share considerable genetic similarity which may suggest their common origin (Fig. 4). This is unexpected considering the fact that LimeStone1 was isolated in Belgium and ϕD3 and ϕD5 were isolated in different regions in Poland. The core (common) genome of ϕD3, ϕD5 and LimeStone1 consists of 178 genes, whereas only 7, 13 and 6 genes are specific for phages ϕD3, ϕD5 and LimeStone1, respectively (Fig. 4).

Interestingly, the majority of the genes found in ϕD3 do not have homologs in T4 (one of the best described and characterized *Myoviridae* bacteriophages) and only two genes are present in both phages *viz*. (i) phage recombination protein and (ii) phage endoribonuclease translational repressor of early genes.

Bacteriophage capsid assembly protein (gp20) was used for phylogenetic analysis as previously described [16, 17]. Nucleotide sequences of gp20 proteins of LimeStone1 (Genbank accession: NC019925), bacteriophage ϕD5 (KJ716335), *Shigella* phage phiSboM-AG3 (NC013693), *Salmonella* phage SKML-39 (NC019910), *Klebsiella* phage 0507-KN2-1 (NC022343), *Salmonella* phage vB SalM SJ3 (NC024122), *Escherichia coli* phage PhaxI (NC0194521), *E. coli* phage vB_EcoM_CBA120 (NC016570), *Salmonella* phage SFP10( NC016073), *Salmonella* phage PhiSH19 (NC019530), *Salmonella* phage Maynard (NC022768), *Salmonella* phage Marshall (NC022772), *E. coli* phage ECML-4 (NC025446), *Salmonella* phage vB SalM SJ2 (NC023856), *Salmonella**p*hage Vi01 (NC015296) were obtained from GenBank. ClustalX was used to align nucleotide sequences and to manually correct aligned sequences prior to further analyses. Phylogeny studies were performed with the use of the Phylip program [18] and Molecular Evolutionary Genetic Analysis (MEGA6) software [19]. Dendrograms were created using the Maximum likelihood method followed by calculating the *p*-distance matrix for aligned gp20 nucleotide sequences (length of gp20 nucleotide sequences: 600 bp, nucleotide substitution model: K80 Kimura) with the bootstrap support fixed to 1000 re-samplings. To root the tree, a gp20 nucleotide sequence from *Enterobacteriaceae* bacteriophage T4 derived from its complete genome (NC000866) was used.

As expected, ϕD3 showed the highest similarity to the other described *Dickeya* spp. bacteriophages (LimeStone1 and ϕD5). On the basis of the gp20 phylogenetic analysis, ϕD3 was also closely related to *Shigella* phage phiSboM-AG3 and *Salmonella* phage SKML-39. The largest phylogenetic distance was observed between ϕD3 and *Enterobacteriaceae* phage T4 (Fig. 3b).

## Conclusions

As far we know, the ϕD3 is the third bacteriophage able to infect (and kill) several species of *Dickeya* that has been genetically characterized in depth and is also the second *Dickeya* spp. lytic bacteriophage isolated in Poland. We expect that the availability of an additional *Dickeya* spp. specific bacteriophage would improve our understanding of bacteriophage – bacteria interactions and gives an insight on conservation and evolution of *Dickeya* spp. lytic bacteriophages as well as improve our knowledge on *Dickeya* spp. ecological fitness in complex (soil, rhizosphere and phyllosphere) environments.
